# *Oldenlandia diffusa* Promotes Antiproliferative and Apoptotic Effects in a Rat Hepatocellular Carcinoma with Liver Cirrhosis

**DOI:** 10.1155/2015/501508

**Published:** 2015-03-10

**Authors:** Yun-Young Sunwoo, Jin-Hee Lee, Ho Yong Jung, Yu Jin Jung, Moon-Seo Park, Yong-An Chung, Lee-So Maeng, Young-Min Han, Hak Soo Shin, Jisoo Lee, Sang In Park

**Affiliations:** ^1^Comprehensive Hospital for Advanced Cancer, International St. Mary's Hospital, College of Medicine, The Catholic Kwandong University of Korea, Incheon 404-834, Republic of Korea; ^2^Department of Pharmacy, College of Pharmacy, Seoul National University, Seoul 151-742, Republic of Korea; ^3^Institute of Catholic Integrative Medicine (ICIM), Incheon St. Mary's Hospital, The Catholic University of Korea, Incheon 403-720, Republic of Korea; ^4^EIT/LOFUS Research Center, International St. Mary's Hospital, College of Medicine, The Catholic Kwandong University of Korea, Incheon 404-834, Republic of Korea; ^5^Department of Radiology, Incheon St. Mary's Hospital, The Catholic University of Korea, Incheon 403-720, Republic of Korea; ^6^Department of Internal Medicine, University of Massachusetts Medical School, Worcester, MA 01655, USA

## Abstract

*Oldenlandia diffusa* (OD) is commonly used with various diseases such as cancer, arthritis, and autoimmune disease. Liver cirrhosis is a predominant risk factor for hepatocellular carcinoma (HCC). Here, we show that the therapeutic effect of OD, which was investigated both* in vitro* and chemically, induced HCC model. OD significantly enhanced apoptosis and antiproliferative activity and reduced migration ability of HCC cells.* In vivo*, OD was treated twice a day for 28 days after confirmed HCC model through 2-[^18^F]-fluoro-2-deoxy-D-glucose (^18^F-FDG) imaging. The survival in OD treated groups was shown to have a greater therapeutic effect than the control group. 28 days after OD treatment, OD treated groups resulted in a significant reduction in tumor number, size, ^18^F-FDG uptake, and serum levels such as alanine transaminase, aspartate transaminase, and alkaline phosphate compared to the control group. Also, proliferated cells in tumor sites by OD were reduced compared to the control group. Furthermore, several rats in OD treated group survived over 60 days and liver morphology of these rats showed the difference between tumor mass and normal tissue. These results suggest that OD may have antiproliferative activity, inhibition of metastasis, and apoptotic effects in chemically induced HCC model and can have the potential use for clinical application as anticancer drug of the herbal extract.

## 1. Introduction

Hepatocellular carcinoma (HCC) is the fifth most common cancer and the second leading cause of cancer death worldwide [1, 2]. HCC can be involved from hepatitis B or hepatitis C, cirrhosis due to alcohol consumption, liver disease such as aflatoxin toxicity, hormonal imbalance, and metabolic disease [3–5]. Various treatment options for HCC are available clinically, which include surgery, chemotherapy, and radiotherapy. However, the patients are at high risk in recurrence and the progressing mechanism of HCC including long-lasting inflammation in hepatocytes leading to cirrhosis has not been fully elucidated [6, 7].

A number of studies have reported the beneficial effect of herbal medicine in diverse diseases such as central nervous system and cancer. Also, many herbal drugs and prescriptions have in fact been used clinically for the treatment of cancer and autoimmune diseases. Several studies reported anticancer activity* in vitro* and* in vivo* using natural herb extract [8–10]. Recently, an extract isolated from medicinal herb such as* Oldenlandia diffusa* (OD) has intrigued interest because of its anticancer and chemoprevention effect on various cancer and normal cells [11, 12].

OD is a well-known medicinal plant used in Korean and Chinese herbal medicine for the treatment of hepatitis, tonsillitis, urethral infection, and malignant tumors of the liver and lung. A number of studies have reported multiple biological activities of the OD such as antitumor, chemopreventive, anti-inflammatory, antioxidant, and proapoptotic effect [13, 14]. However, the mechanism behind the antitumor effect of OD is still largely unknown and has certainly not been evaluated in chemically induced liver cancer. OD is identified to include oleanolic acid (3b-hydroxyolean-12-en-28-oic acid, OA), ursolic acid (3b-3-hydroxyurs-12-ene-28-oic acid, UA), asperuloside (IG1), E-6-O-p-coumaroyl scandoside methyl ester (IG2), and E-6-O-p-coumaroyl scandoside methyl ester-10-methyl ether (IG3). OA has an isomer, UA, which is also a pentacyclic triterpenoid compound. OA and UA are distinguished by the position of methyl group between C19 and C20. It has also been reported that dexamethasone, which is a glucocorticoid hormone that has a structure similar to OA and UA. Although OA and UA have similar structure, OA has effects such that the inhibitory effect of OA on cytochrome P450 is stronger than UA [15]. Also, OA and UA exhibit significant antitumor effect and cytotoxic activity in many cancer cell lines such as liver cancer cells, gastric cancer cells, colon carcinoma cells, and fibrosarcoma cells [11, 16–20].

In this study, we investigated the ability of OD enhanced anticancer effect in apoptotic cell death, inhibition of proliferation, and migration of HCC cell lines. In addition, we were able to demonstrate OD enhanced antitumor effects in regulation of hepatic function, glucose metabolism, and metastasis in chemically induced liver cancer model.

## 2. Materials and Methods 

### 2.1. Preparation of* Oldenlandia diffusa* (OD), Oleanolic Acid (OA), and Ursolic Acid (UA)

The herbal sample of OD Roxb was purchased from an herbal market in Gyeongdong. OD was extracted using reflux extraction equipment in hot water for an hour and concentrated using a rotary evaporator before lyophilization. OA and UA were purchased from Sigma-Aldrich (St. Louis, MO, USA). The compounds were dissolved in DMSO and the final concentration of DMSO in samples was 0.15%.

### 2.2. Cell Culture

Liver cancer cell lines such as huh7 and hepG2 were obtained from the American Type Culture Collection (ATCC; Manassas, VA, USA, http://www.atcc.org). The cells were maintained in Dulbecco's Modified Eagle's Medium (DMEM; Hyclone, Logan, UT, USA). The huh7 and hepG2 cells were supplemented with antibiotics and 10% of fetal bovine serum (FBS; HyClone). Cells were incubated at 37°C in a humidified atmosphere containing 5% CO_2_.

### 2.3. Cell Viability Assay

Cell viability was measured using a Cell Counting Kit-8 (CCK-8; Dojindo Laboratories) allowing sensitive colorimetric assay by using highly water-soluble tetrazolium salt that is reduced by dehydrogenases in living cells to give a colored product (formazan). The cells were seeded in 96- (5 × 10^3^ cells/well) or 24-well plates (2 × 10^4^ cells/well) in medium containing 10% FBS and were incubated for 24 h. Subsequently, cells were treated with medium containing OA (0–100 *μ*g/mL), UA (0–100 *μ*g/mL), and OD (0–500 mg/mL) at 37°C for 24 h. The untreated cells in medium containing 0.15% DMSO were used as 100% viability controls. The cells with CCK-8 were incubated for 2 h and were then measured at 450 nm with a spectrophotometer (Molecular Devices, Sunnyvale, CA, USA).

### 2.4. Scratch-Induced Migration Assay

To investigate the effect of OD, OA, and UA on the migration of HCC cells, a scratch-induced migration assay was performed. The cells were seeded in 6-well plates (1 × 10^5^ cells/well) and maintained in DMEM with 10% FBS. The monolayer cells were scraped by using the tip of a 200 *μ*L micropipette, creating linear streaks 2 mm apart. Cells were washed twice with medium to remove nonadherent cells and were further incubated in fresh medium with 10% FBS containing OD (200 mg/mL), OA (100 *μ*g/mL), and UA (50 *μ*g/mL) for 48 h. Wound closure or cell migration was photographed at 0, 24, and 48 h after wounding, using an inverted microscope equipped with digital camera (Nikon, Tokyo, Japan). The images were analyzed into the Meta-Morph imaging software (Molecular Devices Inc., Downingtown, PA, USA). For statistical analysis, at least three independent scratch wound experiments were used for calculations.

### 2.5. Real Time Polymerase Chain Reaction Analysis

Total RNA was extracted from cancer cells using RNeasy Mini Kit (Qiagen, Valencia, CA). cDNA was synthesized with total RNA and oligo(dT) primer (Invitrogen, Carlsbad, CA) using Omniscript RT kit (Qiagen) for real time polymerase chain reaction (RT-PCR). The cDNA samples were diluted 20-fold with water and were used as templates for RT-PCR with 2× SYBR Green Master Mix and oligonucleotide primers. Quantitative RT-PCR was performed using the MyiQ Single-Color RT-PCR Detection System (TaKaRa, Shiga, Japan). Details of the primers are described in the Supporting Information (Supplementary Table 1) (see Supplementary Materials available online at http://dx.doi.org/10.1155/2015/501508). RT-PCR was conducted as described previously [21]. Standard PCR conditions were 95°C (5 min) and then 40 cycles of 94°C (10 s), 60°C (20 s), and 72°C (15 s), followed by the standard denaturation curve with 0.5°C increment for 70 cycles. The ΔCt method was used to calculate the relative levels of expression. The threshold cycle (Ct) value from triplicate measurements was calculated for the expression of the target gene and normalized to that of GAPDH.

### 2.6. Animal Model

The experimental protocols used in this study were designed according to the animal experimental guidelines established by the Institutional Animal Care and Use Committee of Catholic University Medical School. The HCC model procedure was performed as described previously [22, 23]. To induce HCC model, male Sprague-Dawley (180–200 g) rats received intraperitoneal injections of DEN (50 mg/kg; Sigma, St. Louis, MO, USA) once a week for 16 weeks. We confirmed successful development of HCC through PET/CT scan at the last DEN injection.

### 2.7. Experimental Groups and Therapeutic Efficacy Study

Animals were assigned randomly to one of the following three major groups: (1) one group of rats was treated with distilled water as control group (*n* = 9), (2) the second group was fed with OD (100 mg/kg; *n* = 9), and (3) the third group was fed with OD (200 mg/kg; *n* = 9). To assess the survival of the OD treated animals, rats were randomized (*n* = 9, for each group) after last DEN injection and were treated with OD twice a day for 28 days. We recorded starting from day 0 after OD treatment. Survival was followed for a maximum of 60 days.

### 2.8. PET/CT Imaging and Data Analysis

PET/CT imaging and data analysis were performed as previously reported [24, 25]. Images were taken at 0 and 28 days after the OD treatment with a PET scanner using a General Electric Discovery STE (Waukesha, WI, USA). The rats were deeply anesthetized with ketamine and xyzine and ^18^F-FDG (1.1 ± 0.04 mCi) was injected intravenously into the caudal vein. After 30 min, the rats were taken using a PET/CT scanner and body temperature was kept at 37°C with a heating pad on the scanner bed.

To assess the changes of ^18^F-FDG uptake, regions of interest (ROIs) in the lesioned areas were identified in images of the coronal and horizontal sections. Each ROI value was defined on the coronal tomograms that showed the highest uptake to be in the middle of the tumor and calculated as the averaged nCi/cc after calibration of both liver of insulated areas and muscle in the same images. We expressed the standard uptake value (SUV) in ROIs values.

### 2.9. Liver Function Tests

To test liver function, we examined the serum levels of alkaline phosphatase (ALP; Siemens), aspartate transaminase (AST; Siemens), and alanine transaminase (ALT; Siemens, USA) 28 days after OD treatment. Briefly, the blood samples were collected before sacrifice and centrifuged at 3000 rpm for 20 min. The supernatant was analyzed to quantify the concentration in strict accordance with the manufacturer's protocols.

### 2.10. Macroscopic and Microscopic Observation

Four weeks after the last DEN injection, all group rats (*n* = 5, for each group) were deeply anesthetized with 15% urethane and sacrificed by decapitation. The whole liver tissue was sliced into 2 mm thickness. The tumors were counted by macroscopic examination of the liver through two independent investigators according to the criteria.

### 2.11. Histological Examination

28 days after OD treatment, animals from each group were sacrificed for histological examination. The tissue was cut coronally at a thickness of 4 *μ*m from each rat for a total of three blocks. The paraffin sections were dewaxed in histoclear and rehydrated through a grade alcohol series. The slides were incubated with primary antibody against mouse antiproliferating cell nuclear antigen (PCNA; Millipore, Billerica, MA, USA) at 4°C overnight. Subsequently, slides were incubated in Alexa 546-conjugated goat anti-mouse IgM (Molecular probe, Eugene, Oregon, USA). The slides were counterstained with 4′,6-diamidino-2-phenylindole (DAPI; Sigma-Aldrich) and detected using a Zeiss LSM510 confocal microscope (Carl Zeiss, Jena, Germany).

### 2.12. Statistical Analysis

All data are presented as mean values ± standard deviation from at least 3 independent experiments. Statistical differences between different test conditions were determined using Student's* t*-test. Probability values less than 0.05 were considered statistically significant. Also, the statistical analysis of survival was carried out using a log-rank test.

## 3. Result

### 3.1. Anticancer Effect of* Oldenlandia diffusa* (OD) in Hepatocellular Carcinoma (HCC) Cells

**Figure 1:**
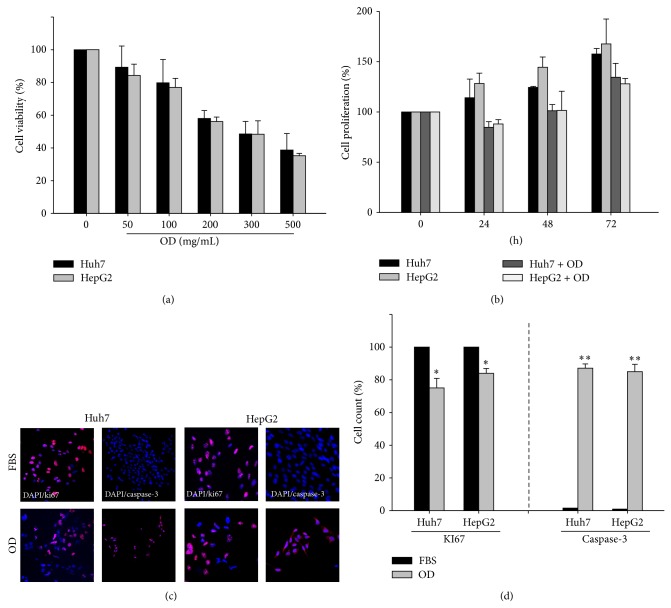
The antitumor effect on hepatocellular carcinoma (HCC) cell lines treated with* Oldenlandia diffusa* (OD). The cytotoxic effect and antiproliferative activity were measured by CCK-8 cytotoxicity assay and immunoreactive cells such as Ki67 and caspase-3. The cytotoxicity effect on HCC cell lines treated with the indicated concentrations of OD (0–500 mg/mL) for 24 h (a). Also, we measured antiproliferative activity of OD (200 mg/mL) against HCC cells for 72 h (b). Ki67 and caspase-3 staining were performed after 24 h in OD (200 mg/mL) ((c), (d); scale bars, 100 *μ*m). OD treatment in huh7 and hepG2 cells reduced ki67-positive cells and increased caspase-3 immunoreactivity. Columns, mean; bar, SE. Student's *t*-test, ^*^ *P* < 0.05 and ^**^ *P* < 0.01.

First, we determined the antitumor effect of OD including cytotoxicity and growth inhibitory effect in hepatocellular carcinoma cells such as huh7 and hepG2. OD treatment exhibited a cytotoxic effect in a dose-dependent manner (Figure 1(a)). Based on these results, we chose OD concentration of 200 mg/mL for the following* in vitro* experiments. We treated HCC cells with OD for 72 h and assessed the number of viable cells. We showed that OD has time dependent growth inhibitory effect (Figure 1(b)). Also, we observed caspase-3 and ki67 through immunostaining (Figure 1(c)). In OD treatment, the activity of ki67-positive cell of HCC cells was decreased and the activation of caspase-3 was significantly increased compared to the control (Figure 1(d)). We questioned what components of OD have antitumor effects in HCC cells. Numerous studies have reported that the principal component in OD including OA and UA could have antitumor effects. We showed that OA and UA have antitumor effects in HCC cells (Supplementary Figure 1). These results suggest that OD has an antitumor effect via the antiproliferation activity and apoptosis of OA and UA in HCC cells.

### 3.2. Suppression of HCC Cell Migration by OD

**Figure 2:**
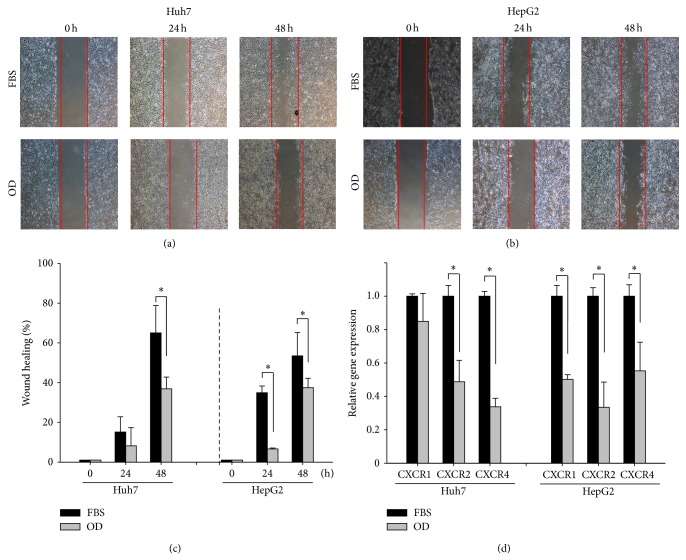
The migration effect of HCC cells by OD. Effect of OD on cell migration by scratch wound migration assay ((a), (b), and (c)). 10% FBS containing DMEM was used as an experimental control. At 48 h after scratching, cell migration is suppressed significantly in huh7 and hepG2 by OD (200 mg/mL) (c). OD influences the expression of migration related receptor including CXCR1, CXCR2, and CXCR4 (d) for 24 h. Columns, mean; bar, SE. Student's *t*-test, ^*^ *P* < 0.05 and ^**^ *P* < 0.01.

To examine the effect of OD on cell migration, we performed the scratch wound healing assay using the huh7 and hepG2 cells. All wound healing images are at the same magnification and time after OD treatment. At 48 h, the wound of FBS treatment was healed approximately 65% ± 13% in huh7 and 53.4% ± 11% in hpeG2 cells from the positive-control cells. OD significantly reduced cell motility in both huh7 (OD: 36.8% ± 6%) and hepG2 (OD: 37.4% ± 4%) compared to the FBS treatment (Figure 2). Furthermore, we showed that OA and UA treatment reduced wound healing (Supplementary Figure 2). We also measured mRNA for migration related receptors such as CXCR1, CXCR2, and CXCR4 in HCC cells by OD, OA, and UA. The expression of migration related receptor was significantly decreased by OD, OA, and UA compared to the FBS treatment (Figure 2(c); Supplementary Figure 2). These results suggested that the OD could inhibit the migration in HCC cells through inhibition of migration receptors by OA and UA.

### 3.3. Survival and Uptake of ^18^F-FDG in HCC Model by OD

**Figure 3:**
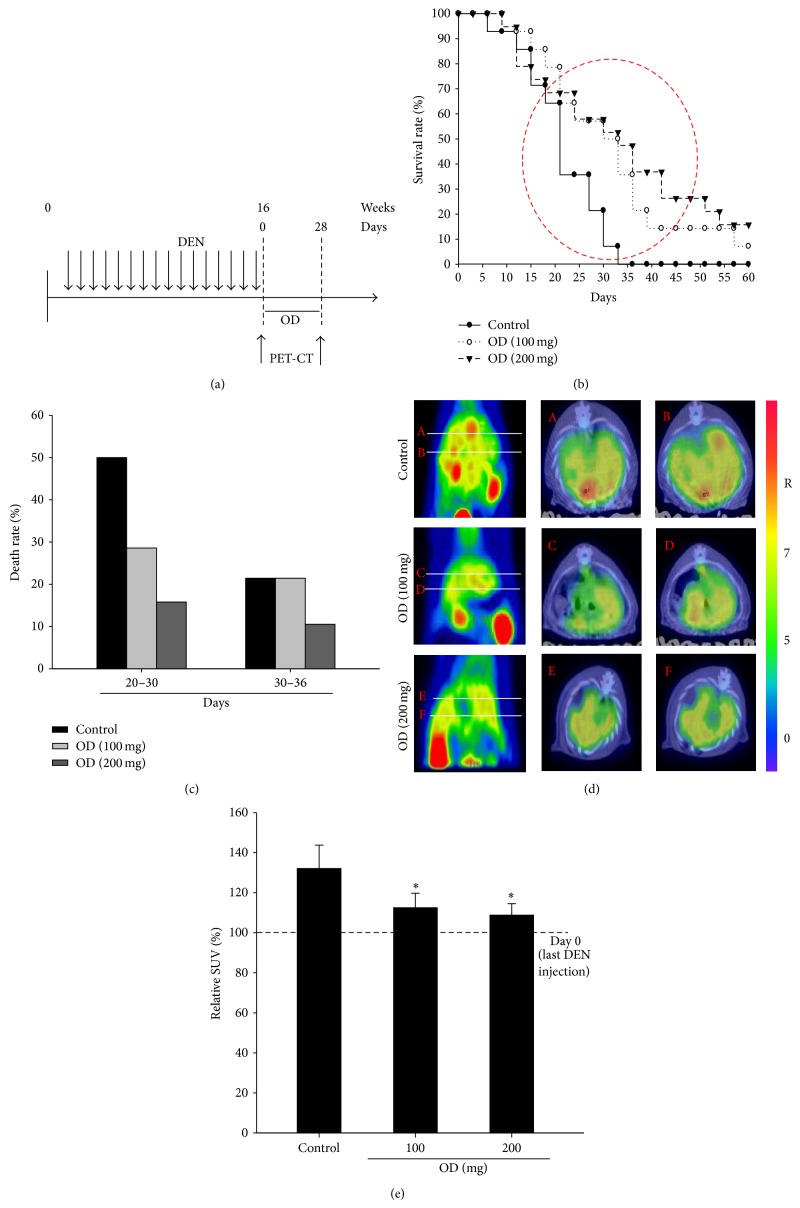
The therapeutic effects of OD for survival and glucose metabolism after HCC. Experimental scheme to test the effect of OD on the survival or ^18^F-FDG uptake in HCC model (a). The survival of HCC was analyzed by a log-rank test based on the Kaplan-Meier method (b). We recorded starting from day 0 after the last DEN injection. We record death rate between 20 days and 36 days (c). ^18^F-FDG PET was scanned at day 0 and 28 days after OD treatment. At 28 days after OD treatment, liver of rats in control group was significantly enhanced uptake of ^18^F-FDG into generated HCC and showed multinodular HCC. However, uptake of ^18^F-FDG on rat's liver in OD (100 and 200 mg) treated group was decreased compared to the control group (d). Quantitative analysis of ^18^F-FDG uptake analyzed at day 0 and 28 days after OD treatment (e). The relative SUV of OD treated group was decreased compared to the control group. Columns, mean; bar, SD. ^*^ *P* < 0.05.

Before treating OD, we confirmed HCC model using the PET/CT imaging. We recorded starting from day 0 before OD treatment and observed the survival rate of rats. 50% of rats in control group died between 20 days and 30 days (Figure 3(b)). The last rat of control group was dead after 36 days. However, the rats of OD treated group (100 and 200 mg/kg) survived more than 40% (Figures 3(a) and 3(b)). Also, 30% of rats in OD treated group (200 mg/kg) survived more than 50 days. PET/CT imaging was performed on day 0 and day 28. ^18^F-FDG has long been demonstrated to be a marker of glucose metabolism in cirrhosis and tumor. The specific areas of liver identified with increased uptake of ^18^F-FDG in PET imaging (Figure 3(c)) and the quantitative image analysis of ^18^F-FDG revealed the SUV (Figure 3(d)). The SUV value of liver in OD treated group (100 and 200 mg/kg) was lower in certain areas compared to the control group (control: 132.0 ± 11.7; OD 100 mg/kg: 112.5 ± 7.2; OD 200 mg/kg: 108.8 ± 5.7).

### 3.4. Reduction for Number of Tumors and Levels of ALT, AST, and ALP in Serum by OD

**Figure 4:**
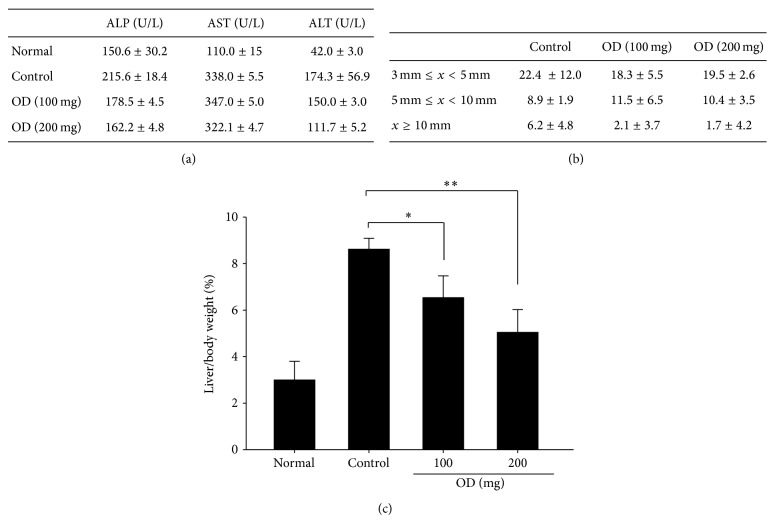
The analysis of level on serum, tumor number categorized by tumor size, and assessment of the liver/body weight 28 days after OD treatment. The blood samples were collected 28 days after OD treatment and the serum level of ALP, AST, and ALT was measured (a). Comparison of tumor number was categorized by tumor size 28 days after OD treatment (b). Also, liver/body weight ratio was measured 28 days after OD treatment. The serum levels of ALP, AST, and ALT in OD treated group were significantly decreased compared to the control group (a). Also, a larger tumor burden in OD treated group was observed less than control group (b). The liver/body weight ratio in rats after OD treatment (c). OD treated group significantly reduced the liver/body ratio compared to the control group. Columns, mean; bar, SD. ^*^ *P* < 0.05 and ^**^ *P* < 0.01.

We investigated whether OD could change hepatic function related factors including ALT, AST, and ALP on serum level in DEN-induced HCC model. We collected serum 28 days after OD treatment and analyzed component of related liver function. Also, a comparison was made by categorizing the tumor number by tumor size and measured the liver/body ratio 28 days after OD treatment. The serum ALP, AST, and ALT levels in OD treated group (100 and 200 mg/kg) were significantly lower compared to the control group (Figure 4(a)). Also, the size distribution of nodules and the liver/body ratio in OD treated group (100 and 200 mg/kg) were significantly reduced compared to the control group (Figures 4(b) and 4(c)). These results suggest that OD may regulate the development and metastasis in tumors by enhancing hepatic function.

### 3.5. Suppression of Tumor Cell Proliferation and Metastasis by OD

**Figure 5:**
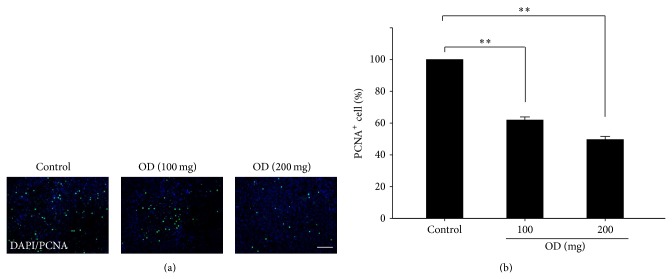
Inhibition of tumor cell proliferation by OD. Histological analysis was shown in PCNA-positive cells 28 days after OD treatment ((a) scale bars, 100 *μ*m). Quantification of PCNA-positive cells in the tumor area (b). The proliferated cells were significantly decreased in the OD treated group (100 and 200 mg/kg) compared to the control group. Columns, mean; bar, SD. ^**^ *P* < 0.01.

**Figure 6:**
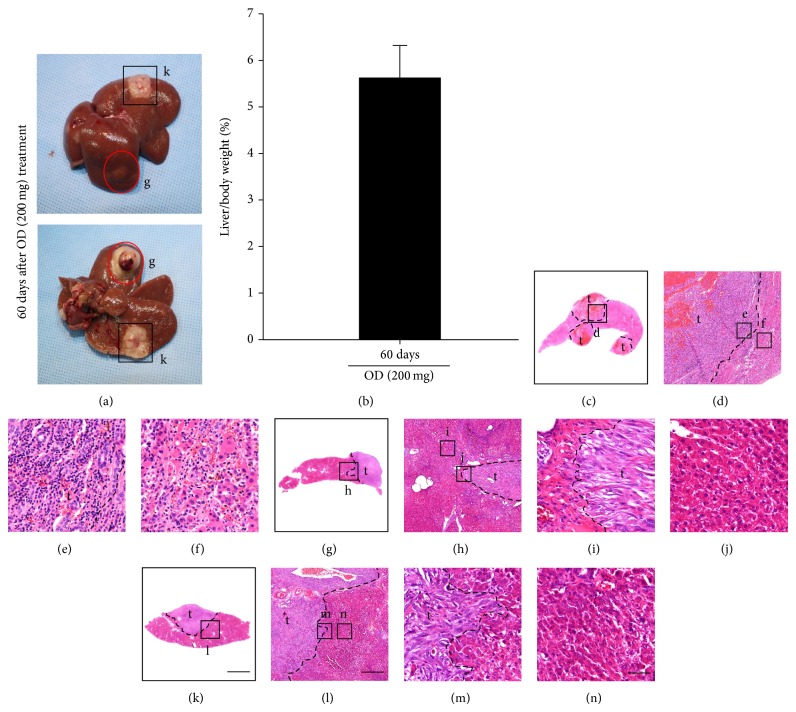
Liver morphology, liver/body ratio, and histopathology analysis 60 days after OD treatment. Liver/body weight ratio was measured after last OD treatment (200 mg/kg). Liver morphology was shown 60 days after OD treatment (200 mg/kg) and H&E staining was done. The large tumor observed in two sites (a). Except for tumor site, the rest of the liver tissue was similar to the normal liver morphology. OD (200 mg/kg) inhibits metastasis of tumor. Liver/body weight ratio in rats 60 days after OD treatment (b). The liver/body ratio after OD treatment (200 mg/kg) was not different between 28 days and 60 days (columns, mean; bar, SD). We show that tumor growth including the malignant nodules over 3 mm of a diameter, cirrhosis, and necrosis in tumor were significantly progressing in slides at 28 days in control group ((c)–(f)). 60 days after OD treatment, we observed the boundary between the large tumor and normal liver cells ((g)-(f), (k)–(m)). Also, no cirrhosis was shown and normal liver cells exist ((j), (n)). Scale bars ((a), (g), and (k)); 5 mm ((d), (h), and (l)); 500 *μ*m ((e)-(f), (i)-(j), and (m)-(n)); 50 *μ*m.

To investigate the inhibitory effect of OD on the proliferation in tumor cells, we performed immunostaining in PCNA, which is a cell proliferation marker. In OD group, PCNA-positive cells were significantly decreased compared to the control group (Figure 5). Also, we observed the hepatic morphology of rats more than 60 days after OD treatment. 60 days after OD treatment (200 mg/kg), the large tumor was observed in two sites (Figure 6(a)). Except for tumor site, the rest of the liver tissue was similar in morphology to the normal liver. We think that this is very interesting result because of numerous developments of multinodular HCC and cirrhosis in chemically induced HCC model. Also, the percentage of liver/body weight ratios in rats 28 days and 60 days after OD treatment (200 mg/kg) was not significantly different (Figures 4(c) and 6(b)). Furthermore, we demonstrated a significant tumor growth including malignant nodules over 3 mm of a diameter, cirrhosis, and necrosis using the H&E staining at 28 days in control group (Figures 6(c)–6(f)). 60 days after OD treatment (200 mg/kg), a boundary line between the large tumor and normal liver cells was observed (Figures 6(g)-6(f) and 6(k)–6(m)), as well as normal hepatocellular architecture (Figures 6(j) and 6(n)). These results suggest that OD (200 mg/kg) may inhibit the ability of cell proliferation and metastasis of tumor through enhanced hepatic function.

## 4. Discussion

Herbal medicine has been used in Asian countries and numerous studies have reported anticancer activity* in vitro* using a natural herb extract [8, 26]. OD, a traditional Chinese and Korean medicinal plant, is known for its anticancer activities [14, 27, 28] and is a valuable medicinal plant for its multipurpose uses. However, the mechanism of antitumor effect of OD is still largely unknown and has not been evaluated in chemically induced liver cancer. In this study, we investigated the anticancer effect of OD on chemically induced liver cancer model. Also, we studied the anticancer effect of OD such as apoptosis, cell proliferation, and migration of HCC cells including huh7 and hepG2 and elucidated possible mechanisms.

This study demonstrated that OD induced apoptosis by caspase-3 pathway in HCC cells. Also, OD suppressed proliferation of HCC cells compared to untreated control cells. These results were consistent with previous findings of anticancer effect in breast cancer cells [14]. Since HCC exhibits a high rate of metastasis, it would be of great importance to understand the migration receptor related gene expression that affects the process of HCC metastasis. We were able to demonstrate the effect of OD in inhibiting cell migration in HCC cells and reducing expression of migration related chemokine receptors such as CXCR1, CXCR2, and CXCR4. Both CXCR1 and CXCR2 are predominantly expressed within the immune system and bind with the highest affinity to the chemokine interleukin-8. CXCR1 and CXCR2 can mediate migration, invasion, and proliferation individually in cancer cells [29]. CXCR4, known as SDF-1*α* receptor, plays a critical role in normal physiology as well as the pathology of many human diseases including cancer, inflammation, and autoimmune disease. Also, CXCR4 correlates with the histological grade and invasive capacity of tumor cells, as well as with tumor cell survival [30]. Our results suggest that OD has anticancer effect in caspase-3 induced apoptosis, antiproliferation, and the inhibition of migration. However, we do not know the exact mechanism of OD as OD consists of numerous compounds. We hypothesized that the principal component in OD including OA and UA could have antitumor effects. The OA and UA are present in OD at high abundance and are pentacyclic triterpenoids that exist widely in plants [31, 32]. OA and UA share similar chemical structure and the only difference of the two compounds is the position of one methyl group between C19 and C20. Our results show OA and UA inhibit cell proliferation of huh7 and hepG2. Also, OA and UA induce apoptosis involving mitochondrial pathway through caspase-3 activation and reduced expression of migration related receptors such as CXCR1, CXCR2, and CXCR4. Our results suggest that OA and UA presented in OD at high abundance can play critical roles of anticancer effects.

We investigated the anticancer effect of OD in* in vivo* model using the chemically induced liver cancer model. We employed a DEN-induced cancer model which can establish HCC and liver cirrhosis simultaneously as the malignant tumor progresses from liver cirrhosis. This model may provide similarity between experimental and human HCC and be a better scheme for studying human HCC than the implanted HCC model with cirrhosis. The survival* in vivo* experiment revealed that OD enhances survival rate compared to the control group. Also, 30% of rats in OD treated group survived more than 50 days. ^18^F-FDG can provide a useful indicator of tumor growth and metastasis because ^18^F-FDG accumulates in tumor mass with increased glucose metabolism [33]. Also, ^18^F-FDG imaging can provide additional information including tumor development and secondary tumor growth. In a previous study, we have reported that we analyzed ^18^F-FDG imaging using the PET/CT time-dependently in DEN-induced liver cancer model. We analyzed ^18^F-FDG imaging at day 0 and day 28. The uptake of ^18^F-FDG of liver in OD treated group was lower in certain areas compared to the control group. Furthermore, OD can change hepatic function related factors including ALT, AST, and ALP on serum level in HCC model. Serum components such as ALT, AST, and ALP are markers of hepatic function and their increases in blood indicate liver damage by the cytotoxic effects of DEN [34, 35].

Our results show that OD may regulate glucose metabolism in tumors through enhancing hepatic function. Also, these results show similar pattern as reduction of the number categorized by tumor size, liver/body ratio, and cell proliferation in tumors. We investigated liver morphology, tumor number, and size of rats that survived more than 60 days after OD treatment. We observed the large tumor in two sites. Except for tumor site, the rest of the liver tissue was similar in morphology to normal liver, despite the chemically induced HCC model generating numerous tumors and cirrhosis in the liver (Figure 4(b)). This is an interesting finding suggesting that OD may have a role in enhancing the hepatic function and inhibiting the metastasis in chemically induced HCC model. In the pathology analysis, we observed normal hepatocellular architecture in the boundary around the tumor site. Also, the liver/body weight ratio in rats 28 days and 60 days after OD treatment was not different. These results suggest that OD inhibits the ability of cell proliferation and tumor metastasis through the regulation of hepatic function, glucose metabolism, and the migration related receptors.

In conclusion, this study demonstrated that OD treatment has anticancer effects in inhibiting the growth of HCC cell in chemically induced liver cancer model. Also, OD can regulate the hepatic function and glucose metabolism. Furthermore, our results have shown that OA and UA derived from OD extracts act as the bioactive components of the anticancer effects. Further studies are needed to identify the detailed mechanisms involved in the therapeutic effect of OD* in vivo*.

## Supplementary Material

The Supplementary Material contains primer sequences of real time-polymerase chain reaction in the expression of migration related receptor including CXCR1, CXCR2, and CXCR4. (Supplementary Table 1). Also, the principal component in oldenlandia diffusa such as oleanolic acid and ursolic acid showed anticancer effect including cytotoxicity, antiproliferation activity (Supplementary Figure 1), and migration ability (Supplementary Figure 2) in hepatocellular carcinoma cells.
