# Supplementary material for: Oldenlandia diffusa Promotes Antiproliferative and Apoptotic Effects in a Rat Hepatocellular Carcinoma with Liver Cirrhosis
Source: Evid Based Complement Alternat Med. 2015 Mar 10;2015:501508. doi: 10.1155/2015/501508 (PMC4379430; doi:10.1155/2015/501508)

Table 1. Primer sequences of real time-polymerase chain reaction (RT-PCR)

Primer	Forward	Reverse
CXCR1	5′-CTGGTGATGCTGGTCATCTTAT-3′	5′-CAAGGTCAGGGCAAAGAGTAG-3′
CXCR2	5′-ACTCATCCAATGTTAGCCCAG-3′	5′-GGTGAATCCGTAGCAGAACAG-3′
CXCR4	5′-AGGGAACTGAACATTCCAGAGCGT-3′	5′-AAACGTTCCACGGGAATGGAGAGA-3′
GAPDH	5′-ACACTCACGCGCATCTTC-3′	5′-CCACTTTACCAGAGTTAAAAGCAG-3′

CXCR1, CXC chemokine receptor 1; CXCR2, CXC chemokine receptor 2; CXCR4, CXC chemokine receptor 4; GAPDH, glyceraldehyde-3-phosphate dehydrogenase.

Supplementary Figure 1.

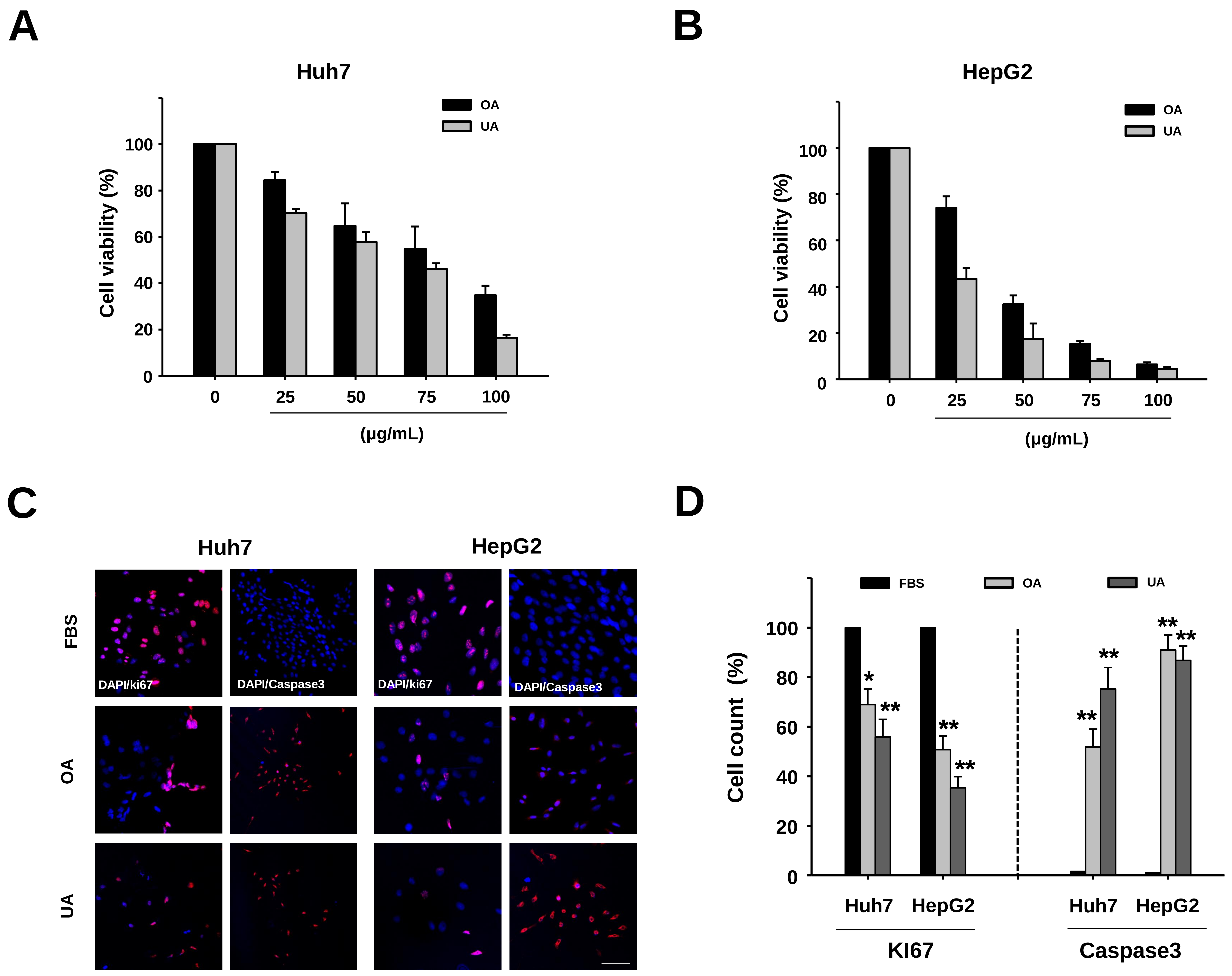

Supplementary Figure 2.

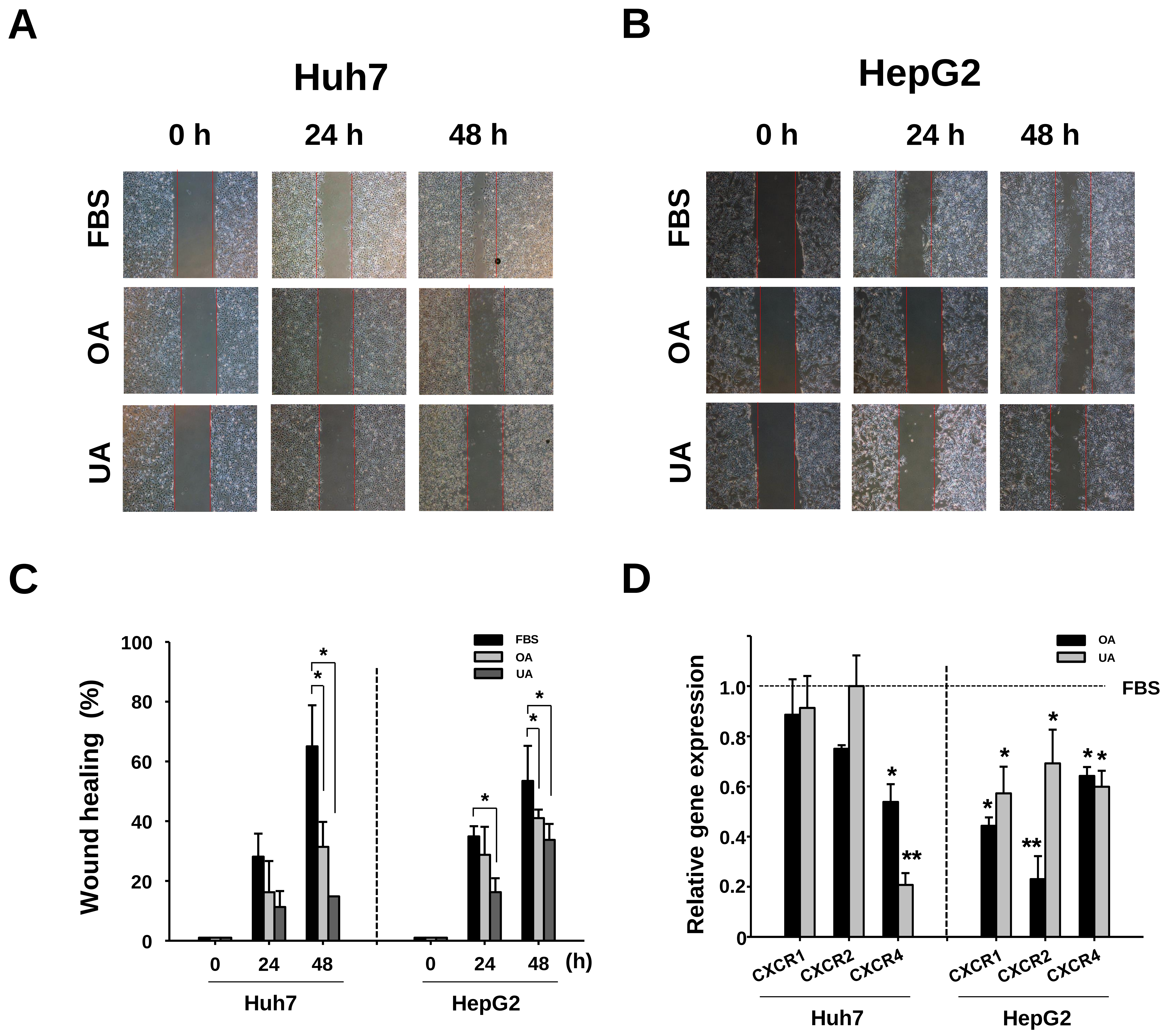

Supplement: Supplementary file 1 — The Supplementary Material contains primer sequences of real time-polymerase chain reaction in the expression of migration related receptor including CXCR1, CXCR2, and CXCR4. (Supplementary Table 1). Also, the principal component in oldenlandia diffusa such as oleanolic acid and ursolic acid showed anticancer effect including cytotoxicity, antiproliferation activity (Supplementary Figure 1), and migration ability (Supplementary Figure 2) in hepatocellular carcinoma cells. [file 501508.f1.pdf]
